# Missouri Dental Journal

**Published:** 1883-05

**Authors:** 


### Missouri Dental Journal.

The publication of this valuable periodical which has been suspended for some months past, has been resumed. It was with regret that we noted its suspension, and it now gives pleasure that the issue is to be resumed, and not only this but the series is to be complete, that is the full complement of numbers will be published to make the series complete.

This has been a favorate journal, and its editor is one of the vigorous and progressive members of the profession, who will be gladly heard each month. May the M. D. J. meet with no interruptions in the future.

